# Association of COVID-19 Vaccination Rates of Staff and COVID-19 Illness and Death Among Residents and Staff in US Nursing Homes

**DOI:** 10.1001/jamanetworkopen.2022.49002

**Published:** 2022-12-29

**Authors:** Soham Sinha, R. Tamara Konetzka

**Affiliations:** 1 Department of Public Health Sciences, Biological Sciences Division, University of Chicago, Chicago, Illinois; 2 Department of Medicine, Biological Sciences Division, University of Chicago, Chicago, Illinois

## Abstract

**Importance:**

It is important to understand the association between staff vaccination rates and adverse COVID-19 outcomes in nursing homes.

**Objective:**

To assess the extent to which staff vaccination was associated with preventing COVID-19 cases and deaths among residents and staff in nursing homes.

**Design, Setting, and Participants:**

This longitudinal cohort study used data on COVID-19 outcomes in Medicare- and Medicaid-certified nursing homes in the US between May 30, 2021, and January 30, 2022. Participants included the residents of 15 042 US nursing homes that reported COVID-19 data to the Centers for Disease Control and Prevention and passed Centers for Medicare & Medicaid Services data quality checks in the National Healthcare Safety Network.

**Exposures:**

Weekly staff vaccination rates.

**Main Outcomes and Measures:**

Main outcomes are weekly COVID-19 cases and deaths among residents and weekly COVID-19 cases among staff. The treatment variable is the primary 2-dose staff vaccination rate in each facility each week.

**Results:**

In the primary analysis of 15 042 nursing homes before the Omicron variant wave (May 30 to December 5, 2021) using fixed effects of facility and week, increasing weekly staff vaccination rates by 10 percentage points was associated with 0.13 (95% CI, −0.20 to −0.10) fewer weekly COVID-19 cases per 1000 residents, 0.02 (95% CI, −0.03 to −0.01) fewer weekly COVID-19 deaths per 1000 residents, and 0.03 (95% CI, −0.04 to −0.02) fewer weekly COVID-19 staff cases. In the secondary analysis of the Omicron wave (December 5, 2021, to January 30, 2022), increasing staff vaccination rates were not associated with lower rates of adverse COVID-19 outcomes in nursing homes.

**Conclusions and Relevance:**

The findings of this cohort study suggest that before the Omicron variant wave, increasing staff vaccination rates was associated with lower incidence of COVID-19 cases and deaths among residents and staff in US nursing homes. However, as newer, more infectious and transmissible variants of the virus emerged, the original 2-dose regimen of the COVID-19 vaccine as recommended in December 2020 was no longer associated with lower rates of adverse COVID-19 outcomes in nursing homes. Policy makers may want to consider longer-term policy options to increase the uptake of booster doses among staff in nursing homes.

## Key Points

**Question:**

Are higher staff vaccination rates associated with lower adverse outcomes of COVID-19 in nursing homes?

**Findings:**

This cohort study of 15 042 nursing homes found that, holding everything else constant prior to the Omicron variant wave, an increase in staff vaccination rates of 10 percentage points was associated with fewer weekly COVID-19 cases among residents, fewer weekly COVID-19 deaths among residents, and fewer weekly COVID-19 cases among staff. During the Omicron wave, increased staff vaccination rates were not associated with lower adverse COVID-19 outcomes.

**Meaning:**

These findings suggest that before the Omicron wave, increasing nursing home staff vaccination rates was associated with fewer COVID-19 cases and deaths among residents and fewer COVID-19 cases among staff.

## Teaser

This cohort study estimates the magnitude to which increasing staff vaccination rates over time was associated with mitigating adverse COVID-19 outcomes among staff and residents in US nursing homes.

## Introduction

The tragic effects of the COVID-19 pandemic in nursing homes have been well established. To date, residents and staff in nursing homes have accounted for approximately 2.16 million cases and close to 155 000 deaths from COVID-19 in the US.^1^ Staff in nursing homes, often risking their own health during the pandemic, provide hours of hands-on care per day and are responsible for maintaining the physical, mental, and psychosocial well-being of residents. Research has shown that higher staffing ratios were helpful in containing outbreaks in nursing homes.^2^ However, studies have also found that more staff traffic between facilities and in and out of areas with high virus prevalence was associated with more cases and deaths in the nursing homes where the staff worked.^3,4^ To mitigate this risk, when vaccines became available in December 2020, staff and residents in nursing homes were among the first to be deemed eligible for vaccination.^5^

States began vaccinating nursing home staff and residents as early as December 14, 2020, and facilities were mandated to report data on vaccination rates from May 23, 2021. The initial data indicated that while the uptake of vaccinations among nursing home residents was in the range of 71.4% to 85.7%, uptake of vaccinations among staff in nursing homes varied substantially, between 31.0% and 82.0%.^6^ Given the wide variation in staff vaccination rates in nursing homes and low rates on average, and given convincing evidence that staff were a primary, if inadvertent, source of COVID-19 outbreaks, a federal mandate requiring vaccinations for all health care workers, including health care workers in nursing homes, was issued on November 5, 2021^7^ (a timeline of vaccination policies is provided in eFigure 1 in Supplement 1).

The evidence base on the effectiveness of the mandate in preventing COVID-19 cases among nursing home residents is weak. One cross-sectional study^6^ in December 2021 found lower levels of COVID-19 cases and deaths among residents in facilities with higher levels of staff vaccination rates during the Delta wave. However, that study was subject to confounding by facility characteristics and could not inform the extent to which increases in vaccination rates over time would lead to improvements in COVID-19 outcomes.^6^ Thus, ongoing policy around staff vaccinations has been made in the absence of a robust evidence base. The present study fills a key gap in the literature by providing estimates on the magnitude to which increasing staff vaccination rates over time were associated with mitigating adverse COVID-19 outcomes among staff and residents in nursing homes in the United States. We also examined the extent to which the 2-dose vaccine regimen for staff in nursing homes continued to be associated with lower adverse COVID-19 outcomes during the Omicron wave of the COVID-19 pandemic.

## Methods

### Data Source and Study Population

In this cohort study, we derived data on weekly staff vaccination rates and weekly COVID-19 cases and deaths among residents and staff from the National Healthcare Safety Network (NHSN). This study was determined to be exempt from review by the Biological Sciences Division of the University of Chicago Institutional Review Board owing to the use of publicly available data. The study followed the Strengthening the Reporting of Observational Studies in Epidemiology (STROBE) reporting guideline.

Although the NHSN now includes data on booster shots, these data are highly incomplete and therefore unusable for our purposes; thus, by *vaccination rates*, we mean only the original COVID-19 vaccination regimens. Using the facility provider identification number from the NHSN files, we merged these data with 3 sources of facility-level data: the Payroll Based Journal,^8^ LTCFocus (Long-term Care Focus),^9^ and Nursing Home Compare archives. The Payroll Based Journal provided data on direct care staff hours per resident day during the study period; LTCFocus provided data on aggregated resident characteristics as of 2018; and the Nursing Home Compare archives provided data on nursing home characteristics as of the last quarter of 2021.

We used county-level identifiers from the NHSN to merge our COVID-19 facility-level data sets with 3 sources of county-level data: County Health Rankings,^10^ the US Census Bureau, and USAfacts.org.^11^ County Health Rankings provided county-level estimates of Black and Hispanic populations in the community and the percentage of the population living without insurance; the US Census Bureau provided county-level estimates of the percentage of the population living in poverty and with Medicaid. USAFacts.org provided data on the prevalence of COVID-19 in the community over time.

For our primary analysis, we restricted our sample to the period after Centers for Medicare & Medicaid Services (CMS) started reporting data on COVID-19 vaccinations in nursing homes (May 30, 2021) and before the Omicron wave of the COVID-19 pandemic (December 5, 2021). To capture the association of staff vaccination rates on COVID-19 outcomes during the Omicron wave of the COVID-19 pandemic, for our secondary analysis we restricted the sample to the period between December 5, 2021, and January 30, 2022. We consider these 2 periods separately because the hypothesized effects of vaccination may differ. Prior to the Omicron wave, the original vaccination regimen (2 doses of BNT162b2 or mRNA-1273 vaccine or 1 dose of JNJ-78436735 vaccine) was shown to be highly effective in reducing risk of infection or adverse outcomes; this same regimen proved to be less effective against the Omicron variant and led to the use of booster shots.^12,13^ For both sets of analyses, we restricted our sample to facilities that reported complete data on staff vaccination rates, COVID-19 cases, and deaths among residents and staff to the Centers for Disease Control and Prevention for all reporting weeks and that passed the CMS data quality assurance checks. Our final analytic samples consisted of 15 042 nursing homes for the primary analysis and 14 879 nursing homes for our secondary analysis (see eFigure 2 in Supplement 1).

### Outcomes

We examined the association of weekly staff vaccination rates with 3 outcomes: weekly COVID-19 cases per 1000 residents, weekly COVID-19 deaths per 1000 residents, and weekly staff COVID-19 cases. Estimation of total residents for resident cases and deaths from COVID-19 was based on the study by Miller et al,^14^ and staff COVID-19 cases were measured as the aggregate number of cases per facility per week. We excluded weekly staff deaths because it was a rare outcome and lacked adequate statistical power for analysis.

### Statistical Analysis

First, we examined unadjusted associations between weekly staff vaccination rates and weekly COVID-19 outcomes in nursing homes in the top and bottom quartile of staff vaccinations. Next, we used multivariable regression models with facility and week fixed effects to determine the adjusted associations between staff vaccination rates and COVID-19 outcomes. Facility fixed effects controlled for all measured and unmeasured time-invariant facility characteristics; by using the fixed-effects design, we essentially used each facility as its own control. Since COVID-19 vaccinations take at least 2 weeks to confer adequate protection against COVID-19^15^ and transmission to residents is likely to take additional time, we estimated lagged effects. For weekly staff COVID-19 cases, we lagged the outcome by 1 week; for weekly COVID-19 cases per 1000 residents, we lagged the outcome by 2 weeks; and for weekly COVID-19 deaths per 1000 residents, we lagged the outcome by 3 weeks in our respective regression models.

We conducted 2 sets of ordinary least-squares regressions for each outcome, 1 with weekly staff vaccination rates measured as a continuous variable, and 1 using quartiles of staff vaccination coverage as a categorical variable, with nursing homes in the lowest 25% of staff vaccination rates as the reference category. In all models, in addition to facility and week fixed effects, we controlled for weekly resident vaccination rates, direct care staff hours per resident day, and county-level prevalence of COVID-19 per 1000 population. Other facility characteristics were absorbed in the fixed effects.

Finally, to explore whether increasing a facility’s vaccination rate was dependent on the absolute level of vaccination and to look for threshold effects, we next used cubic spline regressions with knots at different levels of staff vaccination rates. Cubic splines reflect piecewise linear-adjusted associations between levels of weekly staff vaccination rates and weekly COVID-19 outcomes; that is, they allow the association of additional vaccinations to vary over the distribution of vaccination rates.

As a sensitivity analysis, we used negative binomial regressions to assess the robustness of our results relative to our main approach using ordinary least-squares models. All analyses were performed using Stata, version 17.1 (StataCorp LLC), and statistical significance was determined at 2-sided *P* < .05. Unless otherwise indicated, data are expressed as mean (SD).

## Results

Across our national sample of 15 042 nursing homes, the mean (SD) staff vaccination rate per nursing home during the primary study period between May 30 and December 5, 2021, was 66.05% (20.36%). Among these nursing homes, 9311 (61.9%) were for-profit institutions. The facilities had a mean (SD) of 106.35 (58.85) beds, a mean (SD) of 58.71% (24.13%) residents insured by Medicaid, and a mean (SD) of 17.54% (21.74%) Black residents. Nursing homes had a mean (SD) Nursing Home Care Compare star rating of 3.26 (1.36) stars (range, 1-5, with higher ratings indicating better care). Adjusted mean (SD) total nurse staffing hours per resident-day was 3.98 (0.92). Last, facilities were located in counties with a mean (SD) weekly prevalence of 0.25 (0.49) cases per 1000 population. These counties had a mean (SD) Black population of 11.36% (12.38%) and a mean (SD) Hispanic population of 14.72% (15.42%). Further, sample facilities were located in counties where a mean (SD) 20.13% (6.97%) of the population was covered by Medicaid, 11.35% (5.35%) lacked insurance, and 12.02% (4.28%) lived in poverty (see Table 1).

**Table 1.** Characteristics of Nursing Homes in the Sample

Characteristic	Nursing home data (N = 15 042)^a^
Staff vaccination rate, %	66.05 (20.36)
Nursing home characteristics	
For profit, No. (%)	9311 (61.9)
No. of beds	106.35 (58.85)
Medicaid coverage, %	58.71 (24.13)
Proportion of Black residents, %	17.54 (21.74)
Overall star rating^b^	3.26 (1.36)
Adjusted nurse aide hours per resident	2.35 (0.57)
Adjusted registered nurse hours per resident	0.75 (0.50)
Adjusted LPN hours per resident	0.90 (0.35)
Adjusted total nurse staffing hours	3.98 (0.92)
County characteristics	
Weekly COVID-19 case rate per 1000 population^c^	0.25 (0.49)
Proportion of population, %	
Black	11.36 (12.38)
Hispanic	14.72 (15.42)
Medicaid coverage	20.13 (6.97)
Uninsured	11.35 (5.35)
Poverty	12.02 (4.28)

Abbreviation: LPN, licensed practical nurse.

^a^ Data represent facility-weeks between May 30 and December 5, 2021. Unless otherwise indicated, data are expressed as mean (SD).

^b^ Ratings range from 1 to 5, with higher ratings indicating better care.

^c^ Does not include nursing home cases.

### Unadjusted Associations

**Figure 1.**
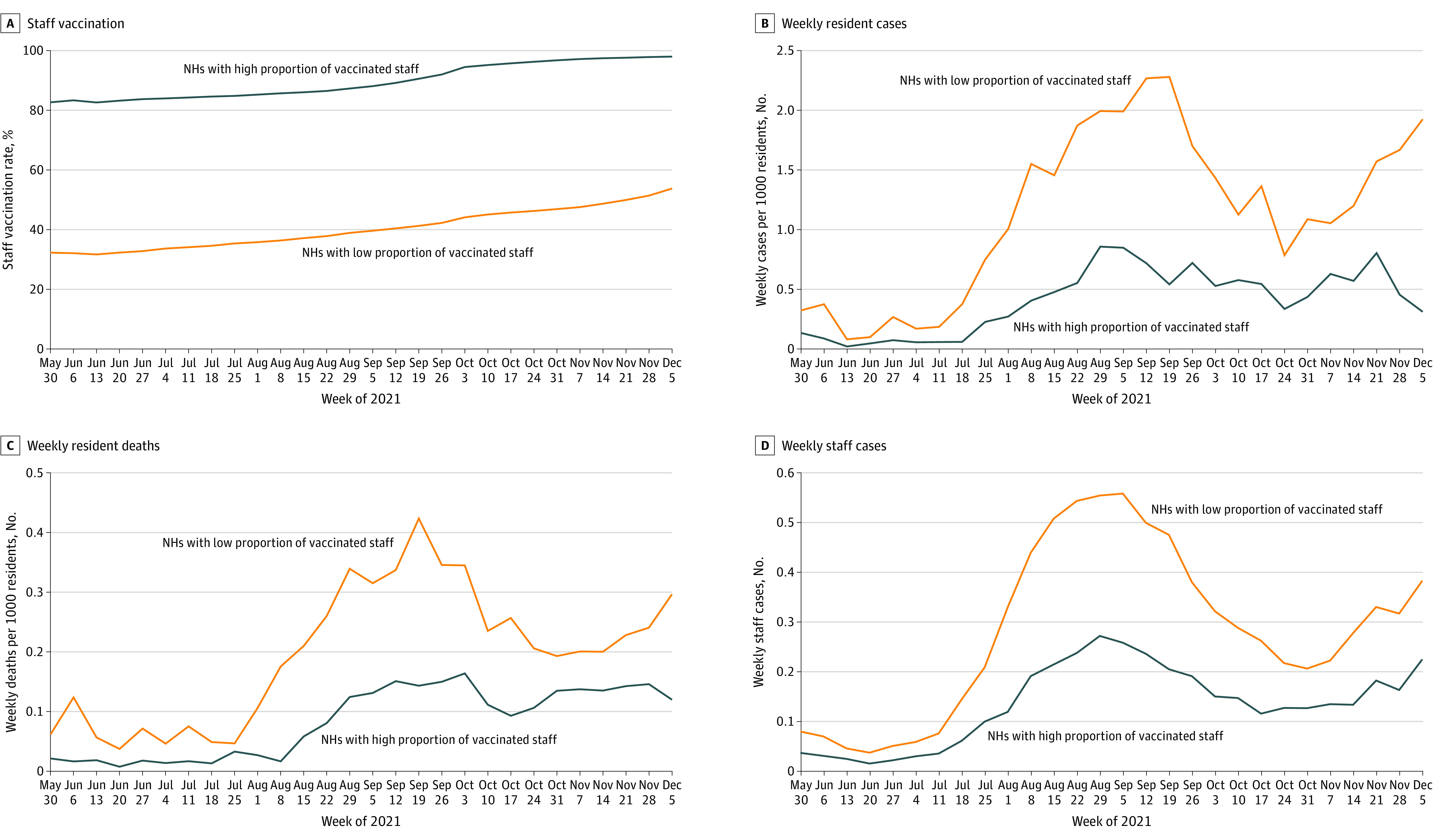
Unadjusted Associations Between Staff Vaccination Rates and COVID-19 Outcomes in Nursing Homes A, Nursing homes (NHs) with a high proportion of vaccinated staff were in the top quartile (highest 25%) of staff vaccination rates; NHs with a low proportion of vaccinated staff were in the bottom quartile (lowest 25%) of staff vaccination rates. B, Quartiles of staff vaccination rates are calculated for each week and thereby vary for each week. C and D, Data represent facility-weeks between May 30 and December 5, 2021.

We stratified our results across quartiles of staff vaccination coverage and found that across the study period, staff vaccination rates increased across all facilities in the country. At the start and end of the study period, mean staff vaccination rate for facilities was 32.28% (8.75%) in the lowest quartile of staff vaccination rates and 82.67% (7.70%) in the highest quartile. By the end of the study period, the mean staff vaccination rate was 53.78% (10.06%) in the lowest quartile and 97.98% (1.93%) in the highest quartile. In other words, vaccination rates increased on average, but substantial variation remained. We also found that compared with facilities in the lowest quartile of staff vaccination rates, facilities in the highest quartile experienced lower levels of weekly COVID-19 cases and deaths among residents and a lower level of weekly COVID-19 cases among staff (Figure 1).

At the onset of the Omicron wave, nursing homes across the US witnessed sharp increases in cases and deaths from COVID-19 among both residents and staff. Facilities across the 2 quartiles of staff vaccination coverage exhibited little to no difference in COVID-19 cases and deaths. However, as the Omicron wave progressed, the differences in cases and deaths (especially among residents) eventually widened between nursing homes across the top and bottom quartiles of staff vaccination rates (eFigure 3 in Supplement 1).

### Regression-Adjusted Associations

Regression-adjusted results from our primary analysis are presented in Table 2. In our first set of regressions—using staff vaccination rates as a continuous variable and controlling for facility fixed effects, week fixed effects, county-level COVID-19 prevalence per 1000 population, resident vaccination rates, and direct care staff hours per resident day—we found that a 10–percentage point increase in the weekly staff vaccination rate on average was associated with 0.13 (95% CI, −0.20 to −0.10) fewer weekly cases per 1000 residents (*P* = .002), 0.02 (95% CI, −0.03 to −0.01) fewer weekly deaths per 1000 residents (*P* = .003), and 0.03 (95% CI, −0.04 to −0.02) fewer weekly staff cases (*P* < .001).

**Table 2.** Association of Staff Vaccination Rates With COVID-19 Outcomes in US Nursing Homes^a^

Variable	Vaccination rates as a continuous variable						Vaccination rates by quartile^b^					
	Cases per 1000 residents^c^		Deaths per 1000 residents^d^		Staff cases^e^		Cases per 1000 residents^c^		Deaths per 1000 residents^d^		Staff cases^e^	
	Coefficient (95% CI)	*P* value	Coefficient (95% CI)	*P* value	Coefficient (95% CI)	*P* value	Coefficient (95% CI)	*P* value	Coefficient (95% CI)	*P* value	Coefficient (95% CI)	*P* value
Staff vaccination rate												
Continuous	−0.01 (−0.02 to −0.01)	.002	−0.002 (−0.002 to −0.001)	.003	−0.003 (−0.004 to −0.002)	<.001	NA	NA	NA	NA	NA	NA
Quartile 1	NA	NA	NA	NA	NA	NA	1 [Reference]	NA	1 [Reference]	NA	1 [Reference]	NA
Quartile 2	NA	NA	NA	NA	NA	NA	−0.31 (−0.57 to −0.04)	.02	−0.05 (−0.09 to −0.01)	.02	−0.06 (−0.08 to −0.05)	<.001
Quartile 3	NA	NA	NA	NA	NA	NA	−0.63 (−0.95 to −0.31)	<.001	−0.08 (−0.14 to −0.03)	.002	−0.12 (−0.14 to −0.09)	<.001
Quartile 4	NA	NA	NA	NA	NA	NA	−0.65 (−1.01 to −0.28)	.001	−0.09 (−0.15 to −0.04)	<.001	−0.13 (−0.16 to −0.11)	<.001
Resident vaccination rate	−0.01 (−0.02 to −0.01)	.001	−0.002 (−0.003 to −0.001)	.003	−0.001 (−0.002 to −0.001)	.01	−0.01 (−0.02 to −0.01)	.001	−0.002 (−0.003 to −0.001)	0.002	−0.001 (−0.002 to −0.001)	<.001
County case rate	0.71 (0.56 to 0.86)	<.001	0.08 (0.05 to 0.11)	<.001	0.13 (0.12 to 0.14)	<.001	0.70 (0.55 to 0.85)	<.001	0.08 (0.05 to 0.11)	<.001	0.13 (0.12 to 0.14)	<.001
Hours per resident day	−0.24 (−0.34 to −0.15)	<.001	−0.02 (−0.03 to −0.01)	.02	−0.004 (−0.01 to 0.002)	.17	−0.24 (−0.34 to −0.15)	<.001	−0.02 (−0.03 to −0.01)	.03	−0.004 (−0.011 to 0.002)	.18

Abbreviation: NA, not applicable.

^a^ Data represent facility-weeks between May 30 and December 5, 2021. Regressions controlled for facility and week fixed effects. Facility fixed effects controlled for confounding from unobserved factors that change across facilities but are constant over time. Week fixed effects controlled for confounding from unobserved factors that change over time but are stable across facilities.

^b^ Quartiles are calculated for each week and therefore vary for each week.

^c^ Weekly COVID-19 cases per 1000 residents are lagged by 2 weeks.

^d^ Weekly COVID-19 deaths per 1000 residents are lagged by 3 weeks.

^e^ Weekly COVID-19 staff cases are lagged by 1 week.

In our second set of regressions with quartiles of staff vaccination rate as categorical variables, we found that staff vaccination rates exhibited a dose-response association with cases and deaths. Using the same set of controls and the 2-way fixed effects, we found that compared with facilities in the lowest quartile of staff vaccination rates, facilities in the highest quartile of staff vaccinations reported fewer cases and deaths among both residents and staff (Table 2). Across all models, the effects were found to be statistically significant.

We also found that county-level COVID-19 prevalence per 1000 population was positively associated with all our outcomes, and direct care hours per resident day and resident vaccination rates were negatively associated with our outcome variables. While the association of county-level COVID-19 prevalence per 1000 population and resident vaccination rate was statistically significant across all the models, the association of hours per resident day was found to be statistically significant only with weekly cases and deaths per 1000 residents (Table 2). All the aforementioned results were found to be in line with the regression models in our sensitivity analysis using negative binomial models (eTable 2 in Supplement 1). In our secondary analysis, we found that during the Omicron wave, staff vaccination rates—both as a continuous variable and using quartiles of staff vaccination coverage—were not associated with COVID-19 cases and deaths in nursing homes (Table 3).

**Table 3.** Association of Staff Vaccination Rates With COVID-19 Outcomes in US Nursing Homes During the Omicron Wave of the COVID-19 Pandemic^a^

Variable	Vaccination rates as a continuous variable						Vaccination rates by quartile^b^					
	Cases per 1000 residents^c^		Deaths per 1000 residents^d^		Staff cases^e^		Cases per 1000 residents^c^		Deaths per 1000 residents^d^		Staff cases^e^	
	Coefficient (95% CI)	*P* value	Coefficient (95% CI)	*P* value	Coefficient (95% CI)	*P* value	Coefficient (95% CI)	*P* value	Coefficient (95% CI)	*P* value	Coefficient (95% CI)	*P* value
Staff vaccination rate												
Continuous	0.03 (−0.05 to 0.11)	.41	−0.001 (−0.007 to 0.005)	.79	0.001 (−0.003 to 0.007)	.57	NA	NA	NA	NA	NA	NA
Quartile 1	NA	NA	NA	NA	NA	NA	1 [Reference]	NA	1 [Reference]	NA	1 [Reference]	NA
Quartile 2	NA	NA	NA	NA	NA	NA	0.64 (−1.05 to 2.35)	.46	0.14 (−0.001 to 0.28)	.06	−0.10 (−0.23 to 0.04)	.16
Quartile 3	NA	NA	NA	NA	NA	NA	−0.31 (−2.56 to 1.93)	.79	0.06 (−0.13 to 0.26)	.52	−0.13 (−0.30 to 0.05)	.17
Quartile 4	NA	NA	NA	NA	NA	NA	−0.63 (−3.48 to 2.22)	.66	−0.01 (−0.22 to 0.21)	.94	−0.27 (−0.50 to −0.04)	.02
Resident vaccination rate	−0.12 (−0.22 to −0.03)	.01	−0.0008 (−0.001 to 0.01)	.90	−0.002 (−0.01 to 0.004)	.51	−0.12 (−0.22 to −0.02)	.01	−0.0005 (−0.01 to 0.01)	.88	−0.002 (−0.01 to 0.01)	.60
County case rate	0.25 (0.08 to 0.42)	.003	0.004 (−0.02 to 0.03)	.70	0.13 (0.11 to 0.15)	<.001	0.25 (0.08 to 0.42)	.004	0.005 (−0.02 to 0.02)	.67	0.13 (0.11 to 0.15)	<.001
Hours per resident day	1.34 (0.84 to 1.86)	<.001	−0.02 (−0.11 to 0.08)	.74	−0.15 (−0.20 to −0.11)	<.001	1.36 (0.85 to 1.88)	<.001	−0.02 (−0.11 to 0.01)	.74	−0.15 (−0.20 to −0.10)	<.001

Abbreviation: NA, not applicable.

^a^ Data represent facility-weeks between December 6, 2021, and January 30, 2022. Regressions controlled for facility and week fixed effects. Facility fixed effects controlled for confounding from unobserved factors that change across facilities but are constant over time. Week fixed effects controlled for confounding from unobserved factors that change over time but are stable across facilities.

^b^ Quartiles are calculated for each week and therefore vary for each week.

^c^ Weekly COVID-19 cases per 1000 residents are lagged by 2 weeks.

^d^ Weekly COVID-19 deaths per 1000 residents are lagged by 3 weeks.

^e^ Weekly COVID-19 staff cases are lagged by 1 week.

### Splines and Piecewise Adjusted Associations

**Figure 2.**
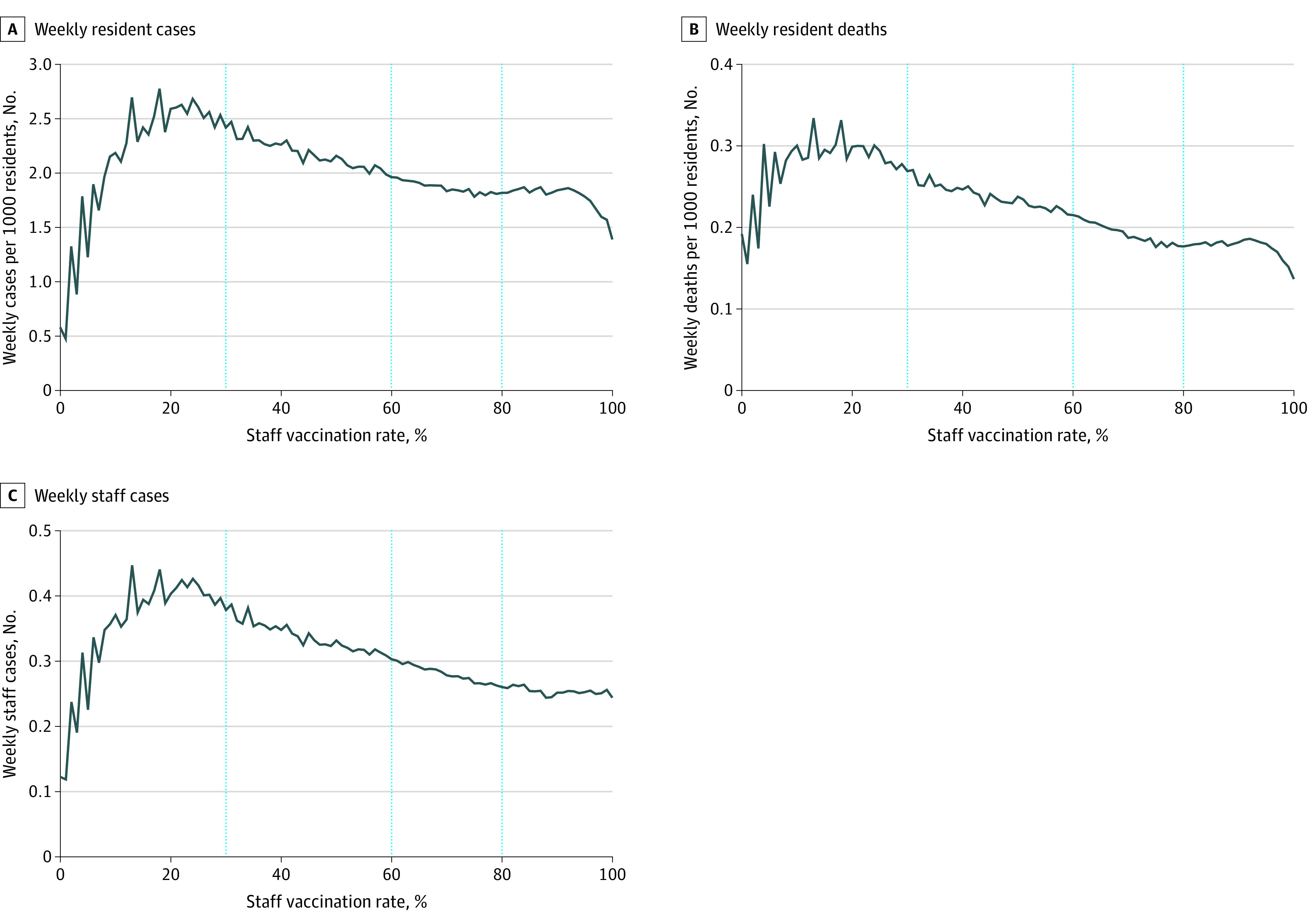
Adjusted Associations Between Staff Vaccination Rates and Weekly COVID-19 Outcomes in Nursing Homes A, Regressions controlled for facility and week fixed effects and adjust for resident, staff vaccination rates, county-level COVID-19 prevalence per 1000 population, and direct care staff hours per resident day. B and C, Data represent facility-weeks between May 30 and December 5, 2021.

We used the distribution of weekly staff vaccination rates across the nursing homes in our sample to identify knots for our spline regressions at 30%, 60%, and 80% of staff vaccination rates. Adjusting for facility and week fixed effects, prevalence of COVID-19 in the community, direct care staff hours per resident day, and resident vaccination rates, we again found that lower staff vaccination rates were associated with higher levels of cases and deaths among both staff and residents, but threshold effects emerged (Figure 2).

Up to a staff vaccination rate of 30%, increases in staff vaccination were associated with worse COVID-19 outcomes (Figure 2). After this point, the direction of the slope changed across all COVID-19 outcomes, indicating benefits of vaccination. The size of the beneficial association then increased with higher vaccination rates. Steeper decreases for COVID-19 cases and deaths among residents and COVID-19 cases among staff were observed at higher staff vaccination levels of 60% and 80%. Compared with weekly resident cases, the threshold effects were more pronounced for weekly resident deaths and weekly staff cases (Figure 2).

## Discussion

We conducted a national examination of the association between staff vaccination rates and COVID-19 outcomes among staff and residents in nursing homes. In line with the findings of previous studies,^6^ the results from our unadjusted analysis showed that COVID-19 cases and deaths were highest among nursing homes with a lower level of staff vaccination rates. Our primary, adjusted analysis found that when controlling for 2-way fixed effects, county-level COVID-19 prevalence, resident vaccination rates, and staffing hours, higher levels of staff vaccination were associated with lower adverse COVID-19 outcomes among staff and residents in nursing homes. Control variables were also significantly associated with the outcomes. The results show a dose-response pattern and an association with lower adverse COVID-19 outcomes at the highest rates of vaccination. Our primary analysis found that highest rates of staff vaccinations were associated with protection to residents and staff amidst both a surging prevalence of virus in the community and a changing landscape of COVID-19–safe practices in nursing homes.

Between May 30 and December 5, 2021, nursing homes across the country on average recorded 27.7 weekly COVID-19 cases per 1000 residents, 3.01 weekly COVID-19 deaths per 1000 residents, and 3760.5 weekly staff COVID-19 cases per week. Per the estimates from our fixed-effects models (see Table 2), a back-of-the-envelope calculation suggests that increasing weekly staff vaccinations in nursing homes on average by even 10 percentage points resulted in 1.98 fewer weekly COVID-19 cases per 1000 residents, 0.3 fewer weekly COVID-19 deaths per 1000 residents, and 401.6 total fewer weekly COVID-19 staff cases.

If we extrapolate to 1 year, our estimates imply that on average, a 10–percentage point increase in staff vaccination rates would have prevented 102.9 COVID-19 cases per 1000 residents, 15.6 COVID-19 deaths per 1000 residents, and approximately 21 000 staff cases nationwide. At the same time, our estimates of the association of staff vaccinations may or may not be generalizable moving forward as new variants of COVID-19 emerge. Our analysis of the beginning of the Omicron wave is consistent with a changing landscape of vaccination effectiveness. Specifically, we found that the original COVID-19 vaccination regimen did not suffice to effectively mitigate adverse COVID-19 outcomes in nursing homes during the Omicron wave of the pandemic. Newer strains of the COVID-19 virus such as Omicron and its subvariants have been found to be more transmissible than the previous strains of the virus. This suggests that going forward, additional booster doses for staff may be needed to effectively manage the pandemic in nursing homes. Although the original vaccination campaign in nursing homes was highly successful in bringing down case and death rates, and mandates led to staff vaccination rates exceeding the thresholds we found for high effectiveness, these policies cannot remain stagnant. As the pandemic evolves, staff vaccination mandates need to evolve as well.

Despite the controversy and debates, COVID-19 vaccinations—as documented herein and in previous studies^6^—seem to have provided some relief for nursing home residents. The introduction of the vaccines was associated with a decline in deaths and cases and made possible new CDC and CMS recommendations that nursing homes fully open to visitors, which provided physical and emotional benefits for nursing home residents.^16^ At the same time, uptake of booster doses among staff in nursing homes is low on average, and there remains substantial variation in the uptake of booster doses across states.^17^ Going forward, policy makers should take note of the existing evidence base on the association of staff vaccination rates with COVID-19 outcomes in nursing homes and use that to guide efforts to optimize policy. Evolving evidence-based policy will be critical.

### Limitations

The study has several limitations. First, we cannot provide a causal estimate of the impact of staff vaccination rates on COVID-19 outcomes among staff and residents. Second, while our study design controlled for time-invariant facility-level factors, we cannot account for potential confounders such as rates of booster shots and adherence to COVID-19–safe practices (eg, handwashing, masking, and social distancing). Third, the CMS started reporting data on staff vaccinations only toward the end of May 2021; therefore, data points from December 2020 to the beginning of May 2021 were excluded from our analysis.

## Conclusions

The findings of this cohort study suggest that increasing staff vaccination rates in US nursing homes was associated with lower adverse COVID-19 outcomes among residents and staff. However, with unpredictable outbreaks of new, more infectious, and transmissible strains of the COVID-19 virus, evolving policy, potentially including mandates for additional booster doses for staff, may be needed to confer adequate protection against adverse outcomes of COVID-19 in nursing homes.

## Notes

Supplement 1. **eFigure 1.** Timeline of COVID-19 Vaccination Policies in Nursing Homes **eFigure 2.** Construction of Analytic Sample **eFigure 3.** Unadjusted Associations Between Staff Vaccination Rates and COVID-19 Outcomes in Nursing Homes (May 30, 2021, to January 23, 2022) **eTable 1. **COVID-19 Outcomes Across Nursing Homes in the Top and Bottom Quartiles of Staff Vaccination Rates **eTable 2.** Effect of Staff Vaccination Rates (SVR) on COVID-19 Outcomes in Nursing Homes: Results From Negative Binomial Regression Models

Supplement 2.
Data Sharing Statement
