# Supplementary material for: Association of COVID-19 Vaccination Rates of Staff and COVID-19 Illness and Death Among Residents and Staff in US Nursing Homes
Source: JAMA Netw Open. 2022 Dec 29;5(12):e2249002. doi: 10.1001/jamanetworkopen.2022.49002 (PMC9856799; doi:10.1001/jamanetworkopen.2022.49002)
Supplement: Supplement 1. — eFigure 1. Timeline of COVID-19 Vaccination Policies in Nursing Homes eFigure 2. Construction of Analytic Sample eFigure 3. Unadjusted Associations Between Staff Vaccination Rates and COVID-19 Outcomes in Nursing Homes (May 30, 2021, to January 23, 2022) eTable 1. COVID-19 Outcomes Across Nursing Homes in the Top and Bottom Quartiles of Staff Vaccination Rates eTable 2. Effect of Staff Vaccination Rates (SVR) on COVID-19 Outcomes in Nursing Homes: Results From Negative Binomial Regression Models [file jamanetwopen-e2249002-s001.pdf]

Supplementary Online Content

Sinha S, Konetzka RT. Association of COVID-19 vaccination rates of staff and COVID-19 illness and death among residents and staff in US nursing homes. *JAMA Netw Open*. 2022;5(12):e2249002. doi:10.1001/jamanetworkopen.2022.49002

eFigure 1. Timeline of COVID-19 Vaccination Policies in Nursing Homes
eFigure 2. Construction of Analytic Sample
eFigure 3. Unadjusted Associations Between Staff Vaccination Rates and COVID-19 Outcomes in Nursing Homes (May 30, 2021, to January 23, 2022)
eTable 1. COVID-19 Outcomes Across Nursing Homes in the Top and Bottom Quartiles of Staff Vaccination Rates
eTable 2. Effect of Staff Vaccination Rates (SVR) on COVID-19 Outcomes in Nursing Homes: Results From Negative Binomial Regression Models

This supplementary material has been provided by the authors to give readers additional information about their work.

© 2022 Sinha S et al. *JAMA Network Open*.

eFigure 1. Timeline of COVID-19 Vaccination Policies in Nursing Homes

December 11,2020:
ACIP recommends the use of vaccinations to contain COVID-19

December 14-22,2020:
States begin vaccinating residents and staff in nursing homes

May 23,2021
CMS starts reporting vaccination rates on the Nursing Home Safety Network (NHSN)

August 18,2021
Initial data show wide variation in the uptake of vaccinations amongst staff in nursing homes

September 09,2021
President Biden remarks that "staff working in nursing homes will be required to get vaccinated"

November 05,2021
CMS introduces Omnibus COVID-19 Vaccination for Health care workers which mandates vaccinations for all staff in nursing homes

November 22,2021
Florida district court denies Florida's request for a preliminary injunction against the mandate

November 29,2021
District court in Missouri enters a preliminary injunction enjoining the mandates to facilities in the 10 states that are plaintiffs

November 30,2021
District court in Louisiana entered a preliminary injunction enjoining the mandate to facilities nationwide

December 15,2021
US Court of Appeals of the 5th circuit upheld the Louisiana court's injunction for the 14 states that were plaintiffs but blocked the ruling for the 26 other states

Mandate goes into effect in the 26 other states

December 16,2021
District court in Texas entered a preliminary injunction enjoining the mandate to facilities in Texas

January 7, 2022
U.S. Supreme Court hears oral arguments in Biden v. Missouri

January 13,2022
U.S. Supreme Court allows CMS vaccine mandate to go into effect

© 2022 Sinha S et al. *JAMA Network Open*.

eFigure 2. Construction of Analytic Sample

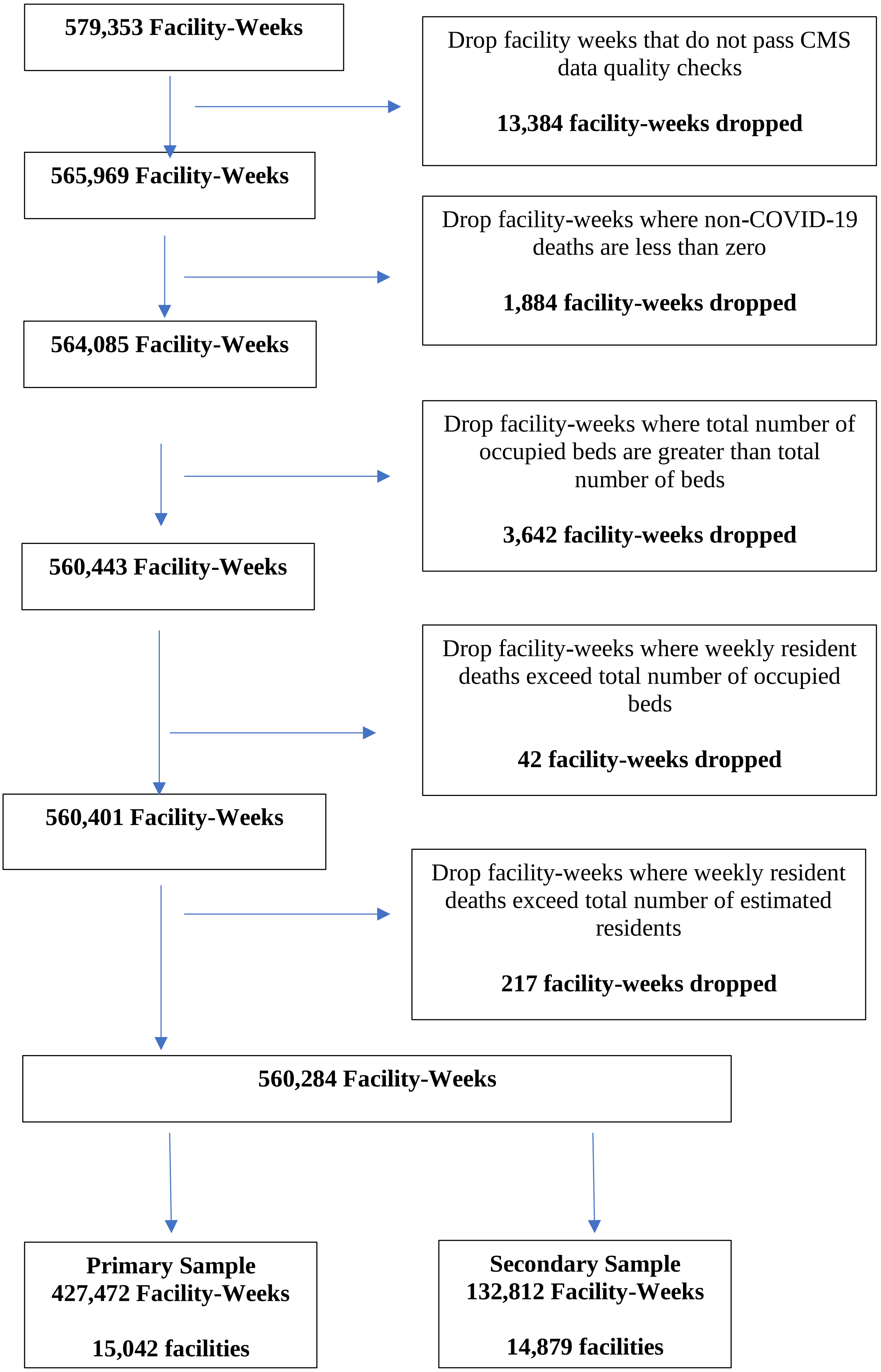

© 2022 Sinha S et al. *JAMA Network Open*.

eFigure 3. Unadjusted Associations Between Staff Vaccination Rates and COVID-19 Outcomes in Nursing Homes (May 30, 2021, to January 23, 2022)

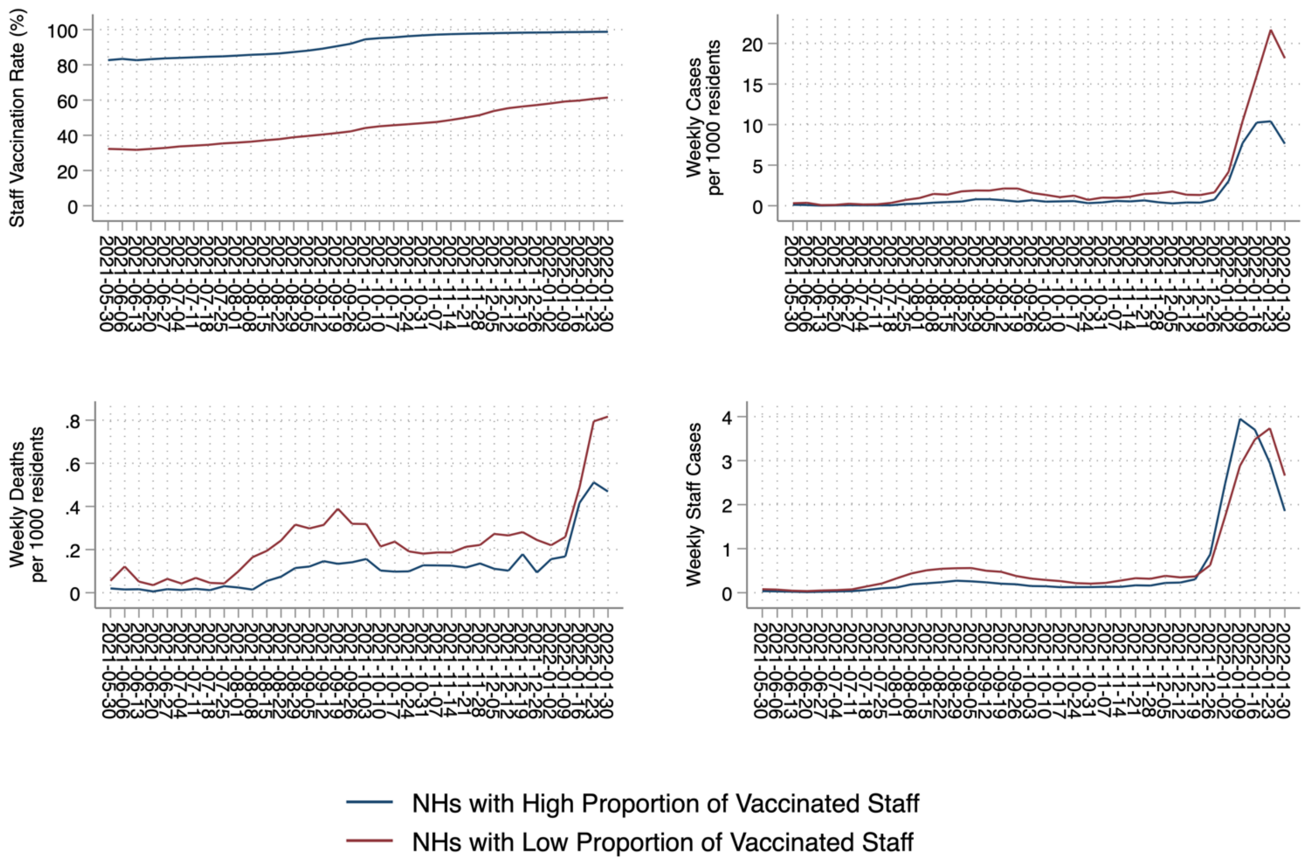

Note: (a) Nursing Homes with high proportion of vaccinated staff refers to nursing homes in the top quartile (highest 25 percent) of staff vaccination rates; nursing homes with low proportion of vaccinated staff refers to nursing homes in the bottom quartile (lowest 25 percent) of staff vaccination rates (b) Quartiles of staff vaccination rates are defined for each week, and thereby vary for each week.

© 2022 Sinha S et al. *JAMA Network Open*.

eTable 1. COVID-19 Outcomes Across Nursing Homes in the Top and Bottom Quartiles of Staff Vaccination Rates

	Cases per 1000 residents		Deaths per 1000 residents		Staff Casess	
**Date**	**Quartile 1**	**Quartile 4**	**Quartile 1**	**Quartile 4**	**Quartile 1**	**Quartile 4**
**5/30/21**	0.323	0.134	0.061	0.021	0.080	0.037
**6/6/21**	0.375	0.088	0.123	0.016	0.070	0.031
**6/13/21**	0.082	0.021	0.056	0.018	0.046	0.025
**6/20/21**	0.099	0.047	0.037	0.007	0.037	0.015
**6/27/21**	0.268	0.074	0.071	0.017	0.051	0.022
**7/4/21**	0.170	0.056	0.045	0.013	0.059	0.030
**7/11/21**	0.186	0.059	0.074	0.016	0.076	0.035
**7/18/21**	0.376	0.060	0.048	0.012	0.144	0.061
**7/25/21**	0.750	0.227	0.046	0.032	0.209	0.100
**8/1/21**	1.006	0.272	0.106	0.026	0.330	0.119
**8/8/21**	1.550	0.406	0.175	0.016	0.439	0.191
**8/15/21**	1.455	0.478	0.209	0.058	0.508	0.215
**8/22/21**	1.873	0.553	0.259	0.080	0.543	0.238
**8/29/21**	1.994	0.858	0.339	0.124	0.554	0.272
**9/5/21**	1.990	0.849	0.315	0.130	0.558	0.258
**9/12/21**	2.268	0.718	0.337	0.150	0.499	0.236
**9/19/21**	2.280	0.541	0.423	0.143	0.475	0.205
**9/26/21**	1.700	0.721	0.345	0.149	0.380	0.191
**10/3/21**	1.433	0.528	0.344	0.163	0.321	0.150
**10/10/21**	1.126	0.578	0.235	0.111	0.288	0.147
**10/17/21**	1.364	0.545	0.256	0.092	0.262	0.116
**10/24/21**	0.786	0.336	0.205	0.105	0.217	0.127
**10/31/21**	1.088	0.436	0.192	0.134	0.206	0.127
**11/7/21**	1.054	0.629	0.200	0.137	0.222	0.135
**11/14/21**	1.199	0.571	0.200	0.135	0.278	0.134
**11/21/21**	1.572	0.804	0.228	0.142	0.330	0.182
**11/28/21**	1.668	0.455	0.240	0.145	0.317	0.163
**12/5/21**	1.925	0.312	0.296	0.119	0.383	0.225

© 2022 Sinha S et al. *JAMA Network Open*.

eTable 2. Effect of Staff Vaccination Rates (SVR) on COVID-19 Outcomes in Nursing Homes: Results From Negative Binomial Regression Models

	**Cases per 1000 residents**	**Deaths per 1000 residents**	**Staff Cases**	**Cases per 1000 residents**	**Deaths per 1000 residents**	**Staff Cases**
SVR	-0.00529***	-0.00437***	-0.00503***			
	(-11.06)	(-3.86)	(-10.70)			
SVR Q2				-0.0687**	-0.123**	-0.101***
				(-3.17)	(-2.66)	(-6.05)
SVR Q3				-0.192***	-0.256***	-0.188***
				(-8.18)	(-4.90)	(-9.57)
SVR Q4				-0.271***	-0.164**	-0.247***
				(-10.65)	(-2.80)	(-10.75)
County Case Rate	0.184***	0.149***	0.195***	0.184***	0.149***	0.196***
	(19.78)	(7.78)	(35.02)	(19.76)	(7.77)	(35.05)
Hours per resident Day	-0.00781	-0.0466*	0.0320***	-0.00807	-0.0478*	0.0319***
	(-0.92)	(-2.31)	(5.25)	(-0.95)	(-2.38)	(5.23)
Facility FE	Yes	Yes	Yes	Yes	Yes	Yes
Week FE	Yes	Yes	Yes	Yes	Yes	Yes

[a] Data represent facility-weeks between December 6th, 2021-January 30th, 2022

[b] Weekly COVID-19 cases per 1000 residents are lagged by 2 weeks

[c] Weekly COVID-19 deaths per 1000 residents are lagged by 3 weeks

[d] Weekly COVID-19 Staff cases are lagged by 1 week

[e] Facility Fixed Effects controls for confounding from unobserved factors that change across facilities but are constant over time

[f] Week Fixed Effects controls for confounding from unobserved factors that change over time but are stable across facilities

[g] * p<0.05; ** p<0.01; *** p<0.001

© 2022 Sinha S et al. *JAMA Network Open*.
