# Supplementary material for: Association of COVID-19 Vaccination Rates of Staff and COVID-19 Illness and Death Among Residents and Staff in US Nursing Homes
Source: JAMA Netw Open. 2022 Dec 29;5(12):e2249002. doi: 10.1001/jamanetworkopen.2022.49002 (PMC9856799; doi:10.1001/jamanetworkopen.2022.49002)
Supplement: Supplement 2. — Data Sharing Statement [file jamanetwopen-e2249002-s002.pdf]

Data Sharing Statement

Sinha. Association of COVID-19 Vaccination Rates of Staff and COVID-19 Illness and Death Among Residents and Staff in US Nursing Homes. *JAMA Netw Open*. Published December 29, 2022. doi:10.1001/jamanetworkopen.2022.49002

Data
Data available: No

Additional Information
Explanation for why data not available: Not Applicable. Data used in the study is publicly available.
